# Response to “Perhaps Fewer, but Faster: Should we Rethink the 90% Stroke Unit Admission Standard?”

**DOI:** 10.1093/esj/aakag059

**Published:** 2026-09-07

**Authors:** Aleš Tomek, Melinda Berg Roaldsen, Bjorn Logi Thórarinsson, Arlene Wilkie, Francesca Romana Pezzella, Hanne Christensen

**Affiliations:** Neurology Department, Second Medical Faculty of Charles University and University Hospital Motol and Homolka, Prague, Czech Republic; Clinical Research Department, University Hospital of North Norway, Tromsø, Norway; Department of Clinical Medicine, The Arctic University of Norway, Tromsø, Norway; Department of Neurology, Landspitali, Reykjavík, Iceland; Stroke Alliance for Europe, London, United Kingdom; Department of Neuroscience, San Camillo-Forlanini Hospital, Rome, Italy; Department of Neurology, Copenhagen University Hospital, Bispebjerg, Copenhagen, Denmark

**Keywords:** admission rate, premorbid disability, stroke unit

We read with great interest the letter by Pensato et al., which raises important considerations regarding the universal application of the ≥90% stroke unit admission target within 24 h in Stroke Action Plan for Europe 2018–2030.^1^ The authors suggest lowering the threshold from 90% and an earlier admission target of 2–6 h.

The purpose of a quality benchmark is not merely to reflect current practice, but to drive improvement. The 90% target is not new; it has been an overarching target of SAP-E since its inception in 2018. Admission to the stroke unit as the first level of care ensures access to time-sensitive interventions and care to prevent complications, including, for example, swallowing tests, which are relevant for all patients with stroke. In this context, a 90% target is valuable precisely because it is ambitious and has not yet been achieved in all but 6 SAP-E countries, according to the latest stroke service tracker data available from 2023. Reducing the target risks, conserving existing limitations rather than incentivising optimisation of the stroke system of care.

Pensato et al.’s argument is that patients at the end of life, with severe preexisting disability or with clearly expressed preferences against intensive intervention, should not be treated in a specialised stroke unit. We fully agree that individualised clinical judgement, particularly in such patients, remains essential and that care should align with patient preferences and goals. But we do not agree with automatically lowering the target below 90%. First, patients with severe pre-stroke disability represent a minority of the overall stroke population in most contemporary datasets. In a large UK multicentre registry cohort of consecutive stroke admissions, the proportion of patients with the most severe premorbid disability was low: only 2.7% had modified Rankin scale (mRS 5) and 6.6% had mRS 4.^2^ Even in patients with severe disability (including mRS 5), quality of life may remain meaningful and should not be dismissed. The disability paradox reminds us that patients may perceive their quality of life as meaningful despite severe disability, and therefore may wish to receive treatment even in situations that clinicians might otherwise consider futile.^3^ In the Czech registry data, 1712 patients with premorbid disability (mRS ≥3) were included, representing a clinically selected cohort treated with reperfusion. Of these, 508 patients had severe premorbid disability (mRS 4–5). Although their likelihood of returning to baseline was lower compared to patients with mRS 3, approximately one-third of patients overall still regained their pre-stroke functional status.^4^ These findings indicate that reperfusion therapy may yield meaningful benefit even in patients with severe premorbid disability and should not be withheld on this basis alone. These data suggest that most patients remain appropriate candidates for stroke unit care, supporting the continued use of a high system-level benchmark.

At the same time, Pensato et al. argue for earlier admission to the Stroke Unit in the first 2–6 h. Access to and timing of reperfusion therapies are monitored using the key performance indicators 7 a, b c and d in the SAP-E project.^1^ It should be emphasised that the 24-h target does not imply that patients should be admitted to a stroke unit only at 24 h; rather, admission should occur within this timeframe, as early as possible, in all patients. It should be noted that in most SAP-E countries, patients are admitted directly to a stroke unit immediately after a stroke, where reperfusion therapy is initiated. However, care should not end there. The discussion appears to reflect a relatively narrow, “ICU-like” interpretation of stroke unit care, primarily centred on reperfusion therapies. This definition is not consistent with the evidence at hand, which extends to mixed acute and rehabilitative stroke units and specialised rehabilitative stroke units. We support this broader We support this broader concept of stroke unit care. The benefits of stroke units extend well beyond acute interventions, as demonstrated by meta-analyses of the original stroke unit trials, which consistently showed the greatest benefit for older and frail patients.^5^ A defining feature of effective stroke unit care is the multidisciplinary team that delivers coordinated, protocol-driven management. This includes early prevention of complications, timely swallowing assessment, early mobilisation and comprehensive supportive care, which underpin the rationale for the 24-h admission window. In addition, stroke unit care should also encompass appropriate end-of-life care when aligned with patient goals, as is standard practice in many healthcare systems, including our own.

To conclude, we believe that maintaining the ≥90% stroke unit (SU) admission target, including the multidisciplinary team in the post-reperfusion period, remains appropriate as a driver of quality improvement across stroke systems of care.
